# Radial extracorporeal shock wave promotes the treatment of non-union of femoral shaft fractures in children: a case report

**DOI:** 10.3389/fsurg.2025.1667385

**Published:** 2025-09-22

**Authors:** He Shang, Tao Ma, Xueqi Liu, Tianxiang Yang, Yongzhu Luo, Jun Li, Jinpeng Liang, Desheng Chen

**Affiliations:** ^1^ Third Clinical Medical College of Ningxia Medical University, Yinchuan, China; ^2^ Department of Joint Surgery, Ningxia Hui Autonomous Region People's Hospital (Affiliated Hospital of Ningxia Medical University), Yinchuan, China; ^3^ Department of Orthopedics, Ningxia Hui Autonomous Region People's Hospital (Ningnan Hospital), Haiyuan, Zhongwei, China

**Keywords:** fracture nonunion, delayed bone healing, fracture, radial extracorporeal shock wave, flexible intramedullary nail, children

## Abstract

**Background:**

Despite the minimally invasive benefits of flexible intramedullary nails (FIN) for pediatric femoral shaft fractures, nonunion may occur. Traditional surgical revisions carry high trauma and risk, while radial extracorporeal shockwave therapy (rESWT) has potential for promoting bone healing but lacks sufficient evidence in such pediatric cases.

**Methods:**

A 4-year-old male with left femoral shaft fracture (AO/OTA 32A2) and FDA-defined nonunion 9 months after FIN fixation received rESWT. Using Gymna ShockMaster 300 (Uniphy), parameters were 6–8 Hz, 2.0–3.0 bar, 1,000–1,500 pulses/week for 3–4 weeks (covering fracture and 2 cm around). Treatment included pre-procedural screening, professional operation, gamified communication, and concurrent rehabilitation.

**Results:**

Post-rESWT, fracture lines blurred then disappeared, with clinical healing achieved and no complications. At 3-month follow-up, intramedullary nail was removed; the patient had 80% weight-bearing capacity, independent walking (mild gait asymmetry), and full weight-bearing recovery later.

**Conclusion:**

rESWT effectively reverses post-FIN nonunion in pediatric femoral shaft fractures, with advantages of non-invasiveness, safety, and good compliance. It is a feasible alternative for surgery-limited cases, though long-term efficacy needs large-scale validation.

## 1 Introduction

Pediatric femoral shaft fractures represent a significant clinical challenge in the field of trauma and orthopedics, often leading to prolonged hospital stays, long-term functional impairments, and asymmetric bone growth (1). Flexible intramedullary nailing (FIN) remains the preferred treatment modality for such fractures due to its minimally invasive nature, ability to preserve blood supply at the fracture site, and facilitation of early functional mobilization (1). However, nonunion may occur in some cases due to anatomical factors, injury severity, unique growth characteristics of children, or suboptimal postoperative management. This complication prolongs the rehabilitation cycle and can result in sequelae such as limb shortening and deformities (2). Traditional salvage measures, such as bone grafting or revision of internal fixation devices, represent secondary interventions with notable risks, including significant surgical complications, high infection rates, and potential physeal injuries, which often reduce acceptance among pediatric patients and their guardians (3). Extracorporeal shock wave therapy (ESWT) has emerged as a noninvasive alternative. Its mechanism of action involves inducing microtrauma at the nonunion site, stimulating periosteal hematoma formation, promoting the release of bioactive growth factors, and rebalancing osteoblast and osteoclast activities, thereby enhancing angiogenesis and accelerating bone healing (4, 5). ESWT is well-established in orthopedics and sports medicine, with proven efficacy not only for fracture nonunion but also for chronic tendinopathies, fascial lesions, and calcified pathologies, supported by robust evidence for its long-term safety and effectiveness (6). For instance, a 5-year follow-up study by Kapusta et al. involving 39 patients with plantar fasciitis demonstrated significant improvement in 91.3% of the ESWT-alone group and complete pain relief with functional recovery in 100% of the ESWT-plus group (7). Adult studies provide a foundation for pediatric applications: the noninvasive nature of ESWT, its favorable side-effect profile (typically manifesting only as minor cutaneous reactions such as petechiae or transient erythema) (8), reports of durable efficacy in tendinopathies, and its mechanism for promoting bone healing collectively suggest its suitability for femoral nonunion in children (9, 10). Crucially, ESWT avoids the trauma of secondary surgery and risks of physeal injury, offering a more minimally invasive therapeutic strategy (11).

**Figure 1:**
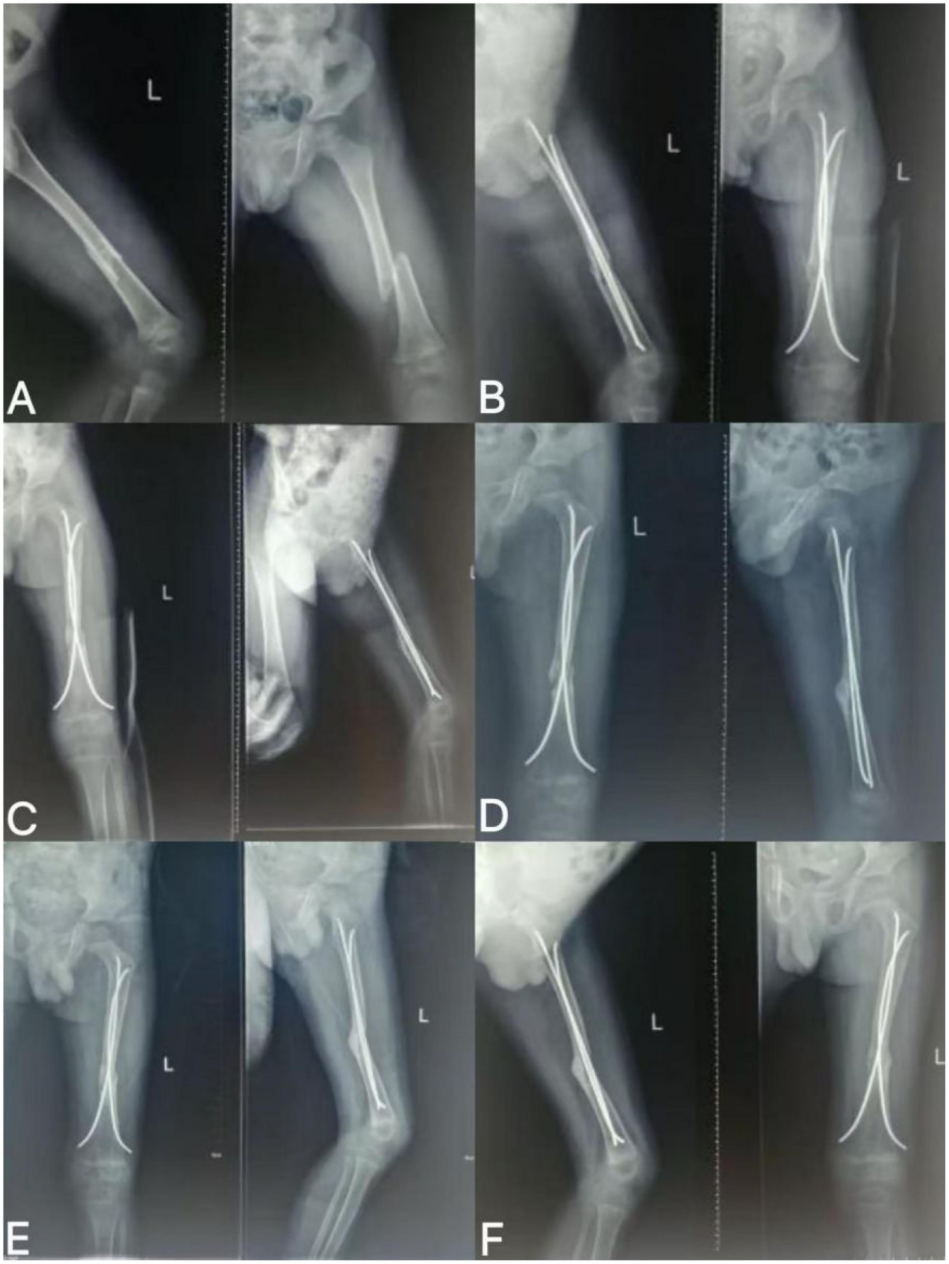
Imaging findings at follow-up after FIN surgery for left femoral shaft fracture. **(A)** Preoperative fracture; **(B)** 1 month after fracture surgery; **(C)** 2 months after fracture surgery; **(D)** 3 months after fracture surgery; **(E)** 5 months after fracture surgery; **(F)** 7 months after fracture surgery.

In clinical practice, shock wave therapy is classified into two primary types: (1) focused extracorporeal shock wave therapy (fESWT) delivers high-energy acoustic waves to the nonunion site, aiming to induce microtrauma and promote osteogenesis through mechanisms such as stem cell activation and angiogenesis (12). This therapy has demonstrated efficacy for nonunion in adult long bones, with outcomes influenced by nonunion morphology (e.g., hypertrophic vs. atrophic) and fracture gap size (13). Adverse reactions like significant hematoma or swelling are rare (14). Although often combined with physiotherapy emphasizing mobility and strength recovery, standardized protocols for pediatric femoral shaft fracture nonunion remain undefined. (2) Radial extracorporeal shockwave therapy (rESWT) employs low-pressure, radially diffused waves and is commonly used for superficial musculoskeletal disorders such as tendinopathies (15). Emerging evidence indicates its potential efficacy in treating superficial bone nonunions, such as scaphoid fractures, with low safety risks (e.g., no reports of hematoma or significant swelling under conventional parameters like 300–400 pulses) (15). Nevertheless, direct evidence supporting rESWT for distal femoral nonunion in children is extremely limited. The radial wave characteristics theoretically allow precise targeting of superficial tissues, potentially offering safety advantages in children due to reduced subcutaneous fat and potentially shallower fracture locations (11). Despite this innovative potential, clinical experience with rESWT for distal femoral nonunion in children remains scarce (15). Moreover, no controlled studies have definitively established the superiority of fESWT over rESWT in the context of fracture healing. The core mechanism of both modalities relies on mechanotransduction pathways (16), providing a theoretical basis for exploring their application in pediatric fracture nonunion.

## 2 Case presentation

The patient, a 4-year-old male, sustained a left femoral shaft fracture (AO/OTA classification 32A2) secondary to trauma from a motor vehicle accident and underwent emergent intramedullary nailing (FIN) fixation. Subsequent serial radiographic evaluations during regular postoperative follow-up demonstrated impaired callus formation, progressive widening of the fracture gap, and disruption of cortical continuity, fulfilling the U.S. Food and Drug Administration (FDA) criteria for nonunion diagnosis—defined as persistent absence of radiographic healing signs for at least 9 months post-injury without progression over three consecutive months (Table 1) (17). Consequently, the parents declined the orthopedic recommendation for revision surgery due to concerns regarding anesthesia-associated risks, surgical morbidity, and economic burdens (18). This preference for nonoperative management was informed by age-related physiological immaturity, which may heighten anesthesia vulnerability and elevate risks of growth disturbances following reoperation (19). Conservative approaches typically entail prolonged immobilization (estimated duration 6–12 months or longer), exhibiting variable efficacy with potential complications including joint stiffness and muscular atrophy (17).

**Table 1:** Patient clinical process timeline chart.

Time node	Key events	Imaging performance	Treatment-related conditions
Preoperative fracture	Fracture of the left femur shaft due to a car accident (32A2）	(Figure 1A) Fracture of the left femoral shaft	Undergoing FIN fixation for emergency femoral fractur
1 Month after fracture surgery	Return to the hospital for the first follow-up	(Figure 1B) shows that the fracture healing tissue begins to grow, but slowly	
2 Months after fracture surgery	Second follow-up	(Figure 1C) The fracture space is slightly widened compared with 1 month after surgery, and the bone cortical continuity is still interrupted	
3 Months after fracture surgery	Third follow-up	(Figure 1D) The fracture healing tissue grows slowly, the fracture space continues to widen, and the bone cortical continuity is interrupted	
5 Months after fracture surgery	Fourth follow-up	(Figure 1E) There was no significant improvement in fracture healing, the fracture space was still widened, and the bone cortical continuity was interrupted	
7 months after fracture surgery	Fifth follow-up	(Figure 1F) The fracture healing tissue grows slowly, the fracture space widening state remains unchanged, and the bone cortical continuity is interrupted	The orthopedic team combined the results of multiple imaging examinations to consider recommending a second revision surgery
9 Months after fracture surgery	Sixth follow-up	(Figure 2B) Slow growth of fracture healing tissue, widening of the fracture space, and disruption of bone cortical continuity meet the FDA definition of bone disunion (Figure 2A) Localization of the left thigh of the child	The child's parents rejected the suggestion of a second revision surgery and hoped for non-surgical treatment

Pertinent medical history revealed no significant preexisting conditions, such as diabetes, hyperthyroidism, or hypothyroidism, which are implicated in altered bone metabolism and nonunion susceptibility (20). There was no personal history of delayed fracture healing or nonunion, and no familial evidence of inherited bone disorders (e.g., osteogenesis imperfecta or osteoporosis), supporting the absence of underlying predispositions to nonunion at this anatomical site (21).

**Figure 2:**
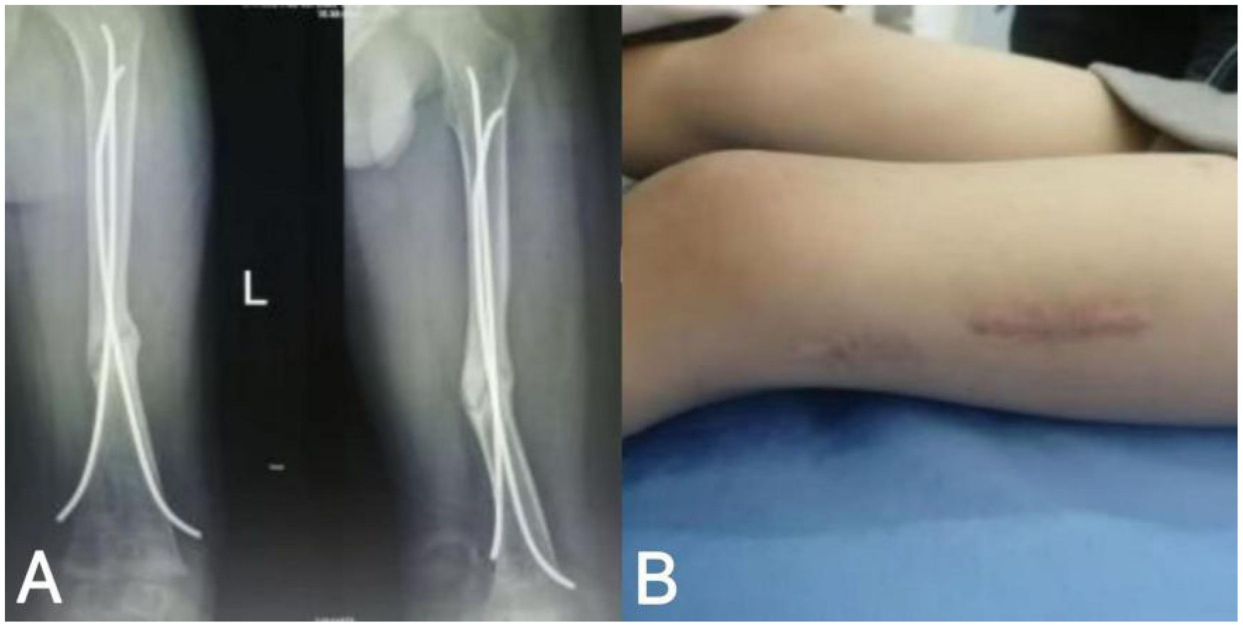
Imaging findings and local skin condition at the affected site prior to rESWT treatment in pediatric patients; **(A)** 9 months after fracture surgery; **(B)** localization of the left thigh of the child.

Physical examination revealed a 6-cm long healed surgical scar in the mid-thigh region of the left leg (Figure 2B), consistent with prior operative intervention for fracture management in the pediatric population. The local skin exhibited normal coloration without elevation in temperature, erythema, or exudation. Palpation demonstrated no significant tenderness; however, axial percussion elicited pain, potentially suggesting underlying complications such as delayed union or nonunion (22). Active range of motion (ROM) of the left knee joint was 0° (extension) to 120° (flexion), while the contralateral limb demonstrated a ROM of 0° to 135° (extension to flexion), indicating mild flexion restriction on the affected side, which may relate to impaired fracture healing or biomechanical factors (23). Muscle strength of the left lower limb, assessed using the Manual Muscle Testing (MMT) scale, was graded as 5 (normal). MMT is a validated method for evaluating muscle function by observing the strength, amplitude, and speed of contraction during active movement against resistance. During testing, the examiner instructed the patient to perform specific movements based on muscle group functions (e.g., knee extension and flexion for quadriceps and hamstrings) while applying appropriate resistance; the ability to resist was graded on a 0–5 scale, with grade 5 denoting full resistance against gravity and maximal force throughout the complete range of motion, signifying unimpaired strength (24). Additionally, superficial and deep sensations were intact, the dorsalis pedis artery pulse was strong, peripheral perfusion and skin coloration were satisfactory, and no indications of neurovascular injury were observed (25).

## 3 Treatment

After a comprehensive assessment of age-related factors, femoral shaft fracture characteristics, and nonunion morphology, rESWT was selected as the primary intervention for this case (17). This decision was based on clinical guidelines and existing research evidence, with its core mechanism involving the stimulation of the bone healing process through mechanical stress (26). rESWT demonstrates significant advantages, including being non-invasive, having high safety, and good patient compliance in pediatric populations (27).

Prior to treatment, nursing staff completed a standardized rESWT contraindication screening questionnaire, covering key information such as the child's medical history and allergy records. The attending physician confirmed the absence of contraindications through clinical examination (28). rESWT was administered by a licensed physical therapist specialized in orthopedic musculoskeletal rehabilitation, who had accumulated over 5 years of clinical experience and participated in 300 fracture rehabilitation cases, ensuring treatment professionalism and safety (29).

**Figure 3:**
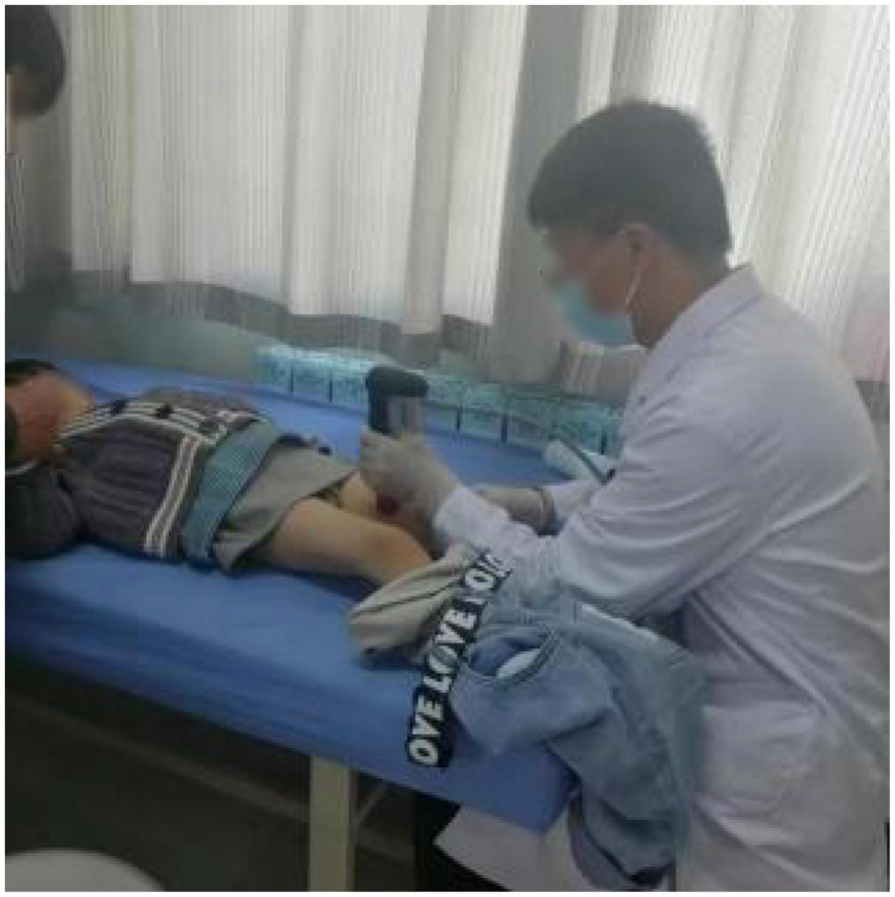
Pictures of children receiving treatment.

Use of rESWT (Gymna ShockMaster 300, Uniphy Elektromedizin GmbH& Co.KG) standardized operating procedures were followed: conductive gel was applied to the fracture site and a surrounding area of 2 cm (30). Parameters were set at a frequency of 6–8 Hz and an intensity of 2.0–3.0 bar (31). Neurovascular avoidance protocols were strictly adhered to minimize risks (28). Treatment started at low intensity and was progressively adjusted based on the child's tolerance (31). Each treatment session delivered 1,000–1,500 impulses, evenly distributed across 3–4 areas (17). Treatment was administered weekly for a total duration of 3–4 weeks (31). During the therapeutic procedure, the child was presented with animated segments from their favorite program. Parents reported that the child maintained complete attention to the animated content throughout the procedure, with no observable signs of crying, resistance, or struggle. Notably, the child exhibited smiling and cooperative behavior during engaging segments of the animation. This gamification approach and animated distraction successfully prevented the child from establishing an association between the therapeutic intervention and distress or fear, thereby substantially reducing treatment-related apprehension. Parental reports indicated complete suppression of typical distress responses such as crying or resistance, with the child instead demonstrating engagement and compliance throughout the medical procedure (Figure 3).

**Figure 4:**
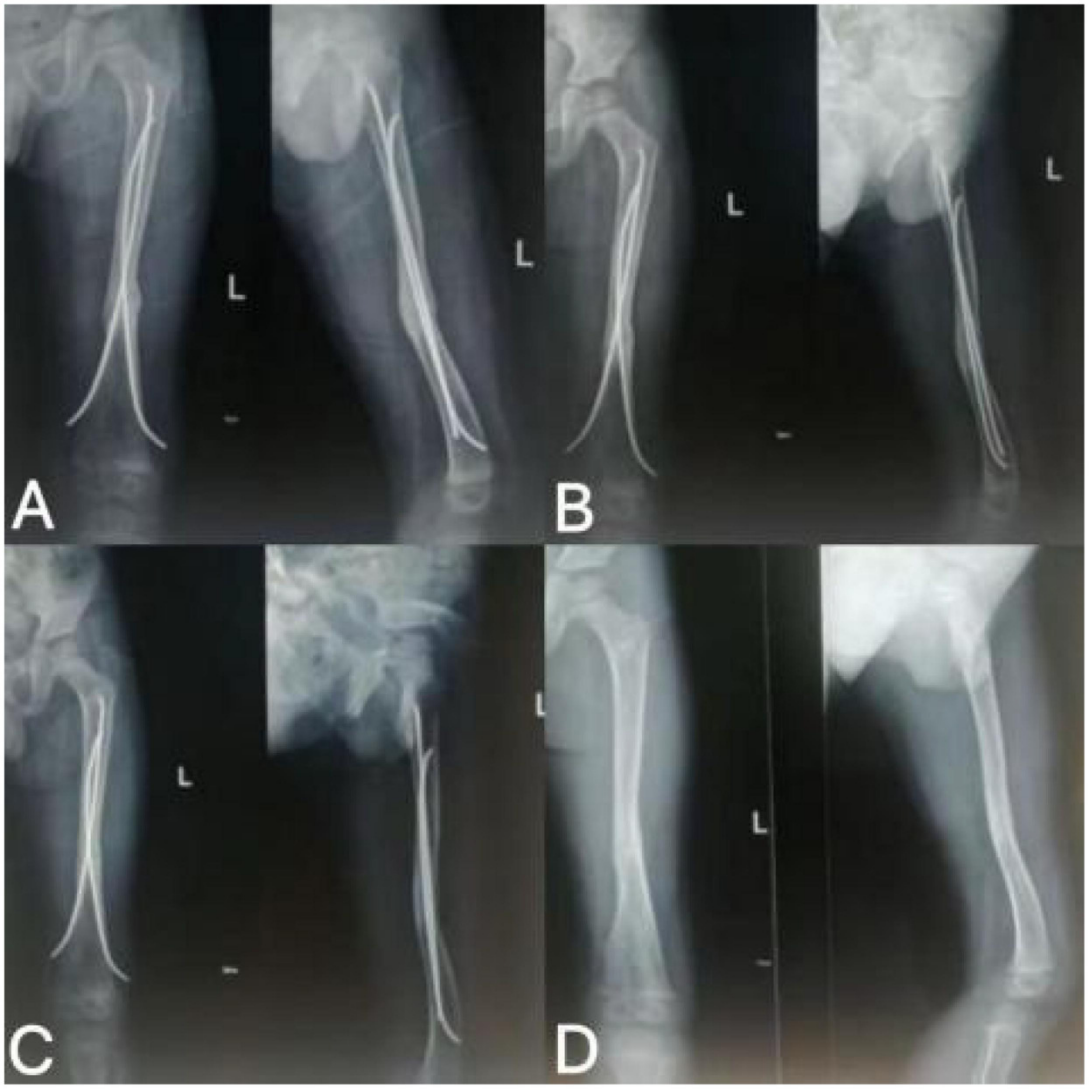
Follow-up imaging findings after rESWT treatment. **(A)** rESWT after 3 sessions of treatment; **(B)** rESWT after 7 treatments; **(C)** rESWT after 8 sessions of treatment; **(D)** Removal of intramedullary nail 13 months after fracture surgery.

During the treatment period, a multidimensional assessment system was established: pain intensity and functional mobility were assessed every two weeks, and radiological follow-up (x-ray examination) was conducted monthly (25). rESWT parameters were dynamically optimized based on assessment results (31). Simultaneously, a systematic rehabilitation regimen was implemented, including daily passive range of motion exercises 3–4 times per day for 10–15 min each, to prevent joint stiffness. High-impact activities and weight-bearing activities were strictly prohibited (23). Medication intervention followed a minimization principle; acetaminophen was used only when necessary for symptom relief, with the patient requiring it only twice during the initial pain phase. Additionally, the patient received three adjunctive physical therapy sessions per week. No other physical therapy modalities, comorbidities, or medications affecting bone metabolism were used.

The child exhibited a consistent trend of gradual improvement following the initial rESWT. At the 13-month follow-up visit, no abnormalities were observed in the child, and the previously implanted intramedullary nail was successfully removed. This outcome signifies that fracture healing and functional limb recovery both achieved an optimal state, with the overall positive trend maintained throughout the treatment and follow-up period (Table 2). Postoperative rehabilitation employed a phased weight-bearing progression: complete non-weight-bearing in the first postoperative week, beginning partial weight-bearing (10%–20%) at 2 weeks, and transitioning to full weight-bearing after 3 months (32). Radiological assessments at all time points confirmed successful bone healing. No adverse events related to rESWT, such as local bruising or exacerbated pain, were observed. The main limitation of this study lies in the lack of validated assessment tools for measuring range of motion or pain intensity (25).

**Table 2:** rESWT post-treatment timeline chart.

Treatment/Follow-up Phase	Imaging Findings	Intervention measures	Functional Status of the Pediatric Patient
After the first rESWT treatment	The x-ray reveals a blurred fracture line (Figure 4A).		
During subsequent treatment	The fracture line has completely disappeared (Figures 4B,C).	Discontinue rESWT treatment and transition to conservative home management: 1. Progressive weight-bearing exercises 2. Adjust daily activities 3. Calcium and vitamin D supplementation	1. Achieved 80% weight-bearing capacity with single-crutch assistance 2. Wears protective brace during outdoor activities 3. Can walk independently, though gait exhibits slight asymmetry
At the 13-month follow-up	No abnormalities observed; intramedullary nail successfully removed (Figure 4D).	Home-based Conservative Management Plan (e.g., rehabilitation exercises, nutritional supplementation)	No abnormalities were detected. The patient's recovery remains stable following removal of the intramedullary nail.

Future studies should incorporate validated tools, such as the Radiographic Union Score for Tibial fractures (RUST) adapted for femoral applications, to quantitatively monitor healing progression and enhance the reliability of outcomes (32).

## 4 Discussion

This case report documents the successful reversal of nonunion in a pediatric femoral shaft fracture initially managed with FIN using rESWT. This outcome provides preliminary support for rESWT as a valuable non-invasive alternative therapy in pediatric fracture management. The discussion below integrates these findings with the current literature, exploring potential mechanisms, comparisons with existing evidence, therapeutic advantages, limitations, and future directions.

The successful application of rESWT in this case can be attributed to its multiple biophysical effects, consistent with the mechanisms described in the existing literature (33). Primarily, shock waves exert mechanical stress on the fracture site, inducing localized micro-trauma and activating the body's inherent trauma repair cascade (25). Specifically, rESWT significantly promotes the expression of vascular endothelial growth factor (VEGF), enhancing local blood circulation and providing essential nutritional support for osseous tissue (26). Furthermore, rESWT stimulates osteoblast activity and enhances callus formation and bone remodeling (17). This stimulation includes enhancing the proliferation, differentiation, and migration capacity of stem cells, directly or indirectly promoting osteogenic differentiation (26), which explains the accelerated bone union observed in this case (17). Additionally, the non-invasive nature of rESWT avoids the trauma associated with revision surgery, a critical advantage in the pediatric population by reducing the risk of iatrogenic periosteal damage or infection inherent in surgical procedures (31).

The findings in this case are consistent with the existing literature on the application of rESWT for fracture nonunion (11). Studies indicate that rESWT serves as a non-surgical alternative to traditional interventions such as bone grafting, demonstrating particular potential in the treatment of adult long bone fracture nonunions, with complication rates typically ranging between 5% and 10% (11). Although surgical intervention remains the standard treatment, it carries a high risk of complications (17), while rESWT provides a safe and minimally invasive alternative (34), which aligns entirely with the outcomes observed in this case. Furthermore, rESWT has significant effects in improving patient pain perception and limb function (11). This advantage not only enhances patient compliance but also provides strong support for reports on fracture healing.

However, this study has two significant limitations that may impact the comprehensiveness, persuasiveness, and generalizability of the conclusions. Firstly, the evaluation of treatment efficacy lacks objective quantitative indicators. Specifically, the study did not utilize standardized tools such as goniometers for an objective assessment of joint range of motion (ROM), which is a core indicator of functional recovery in adjacent joints post-fracture healing; relying solely on clinical subjective observations cannot precisely demonstrate rESWT's actual impact on joint motor function. Additionally, standardized tools like the Visual Analog Scale (VAS) or Numerical Rating Scale (NRS) were not employed to quantitatively document patients' pain levels before and after treatment; pain changes were assessed via oral descriptions instead, resulting in a lack of uniform standards and comparable data that impedes objective verification of analgesic efficacy. Moreover, no gait analysis was conducted using motion capture systems or pressure-sensing devices, preventing evaluation of metrics such as step frequency, stride length, and plantar pressure distribution; consequently, it is impossible to objectively reflect the restoration of weight-bearing capacity, balance function, or ambulation, leading to an incomplete assessment dimension and limiting cross-study comparability. Secondly, as emphasized in one study (35), there is currently no standardized consensus on rESWT treatment parameters (e.g., energy levels, frequency), implying that the efficacy observed in this case may be influenced by specific factors like treatment duration. Failure to standardize these parameters rigorously makes it difficult to exclude uncertainty from variations and hinders replication or verification in subsequent research.

In this case, rESWT demonstrated several notable advantages. Firstly, its noninvasive nature effectively avoided the trauma of revision surgery in the child while significantly reducing risks of periosteal damage and infection (36). Secondly, the treatment is suitable for outpatient settings, and its short duration per session minimized disruption to the child's daily life and preschool activities; strategies such as gamified communication further enhanced treatment compliance (36). Additionally, the high safety profile observed, with no reported significant complications, fully aligns with the current clinical trend favoring non-surgical approaches (34). Notably, as treatment progressed, the child gradually regained ambulatory ability. Parents reported that this outcome not only alleviated their anxiety but, by averting a second surgical intervention, substantially improved their satisfaction with the treatment plan and reduced the psychological burden associated with potential reoperation.

Although this case preliminarily validates the effectiveness of rESWT for pediatric femoral shaft nonunion, significant limitations exist. As a single case report, the findings lack the support of large sample sizes and control groups, limiting generalizability (31). Secondly, research on rESWT is often constrained by heterogeneity in treatment parameters (e.g., energy levels, pulse count) (28), and this case did not fully quantify the impact of such variations. Finally, potential underlying metabolic deficiencies in children (e.g., vitamin deficiency) were not explored as part of a synergistic intervention strategy.

Future research should prioritize the following directions: Firstly, designing multicenter randomized controlled trials (RCTs) to validate the long-term safety and efficacy of rESWT in the pediatric population (23). Secondly, standardizing rESWT indications and contraindications (e.g., poor skin condition, excessive fracture gap) to avoid potential complications (17). Thirdly, exploring combination therapies utilizing drug delivery systems, such as shock wave-responsive nanoparticles, to enhance targeted treatment (26). Finally, establishing standardized protocols for rESWT in pediatric fracture management necessitates robust long-term follow-up incorporating thorough imaging assessments (e.g., changes in bone mineral density) (25).

## 5 Conclusion

In summary, rESWT demonstrated considerable potential in treating pediatric femoral shaft nonunion in this case. Its non-invasive profile, high compliance rates, and biostimulatory effects provide a novel approach for patients where traditional surgery is undesirable or contraindicated. This aligns with the growing trend towards non-operative management and underscores the importance of optimizing fracture healing strategies in children. Based on accumulating evidence (27), we propose rESWT as a promising alternative therapy for managing fracture nonunion. However, optimizing its clinical translation requires further standardization of parameters and large-scale, robust clinical studies. Ultimately, the adoption of rESWT should involve individualized risk-benefit assessments to improve the long-term prognosis of pediatric fracture patients (27).

## Data Availability

The original contributions presented in the study are included in the article/Supplementary Material, further inquiries can be directed to the corresponding authors.
